# Comparison of Immediate Blanket Treatment versus a Delayed Pathogen-Based Treatment Protocol for Clinical Mastitis Using an On-Farm Culture Test at a Commercial German Dairy Farm

**DOI:** 10.3390/antibiotics11030368

**Published:** 2022-03-09

**Authors:** Stefan Borchardt, Wolfgang Heuwieser

**Affiliations:** Clinic of Animal Reproduction, Freie Universitaet Berlin, 14163 Berlin, Germany; w.heuwieser@fu-berlin.de

**Keywords:** clinical mastitis, selective treatment, Accumast

## Abstract

The objective of this study was to compare immediate intramammary antimicrobial treatment of mild and moderate cases of clinical mastitis (CM) with a selective treatment protocol based on on-farm culture results using Accumast^®^. The study was conducted at a 2600 cow commercial farm in Northeast Germany. Using a randomized design, mild and moderate clinical mastitis cases were assigned to either the blanket therapy (BT) or pathogen-based therapy (SELECT) group. Overall, 468 cases were used for final analyses (BT = 236; SELECT = 232). The percentage of cases assigned to the blanket and pathogen-based groups that received intramammary therapy were 100 and 69.9%, respectively. Implementation of a pathogen-based treatment protocol for mild and moderate CM cases resulted in no significant difference in post-event milk production, somatic cell count, survival to 30 d, and days spent in the hospital compared with a blanket therapy protocol. Cows in the SELECT group had reduced odds of being culled within 60 d post CM (odds ratio = 0.54; 95% CI = 0.31–0.93; *p* = 0.027). The use of a pathogen-based treatment protocol using an on-farm culture system has the potential to efficiently reduce antimicrobial use without negative effects on health.

## 1. Introduction

The majority of antimicrobial treatments in dairy cows are administered for the treatment of clinical mastitis. In total, 10.9 million antibiotic treatments were conducted in Germany in 2015 related to mastitis (Wallmann, 2016 personal communication). Most of these treatments are not based on specific etiological information [1]. Instead, the current practice on most farms is to treat all cases of clinical mastitis immediately after diagnosis (i.e., “blanket treatment”) with intramammary antimicrobials. Non-specific use of antimicrobials on farms usually influences the perception of antimicrobials as being misused. Consumers and regulators often associate use of antimicrobials on farms with increasing antimicrobial resistance. Selective therapy for clinical mastitis using on-farm culture results can replace the routine blanket use of antimicrobials in mild and moderate clinical mastitis cases [2]. In addition, on-farm diagnostics avoids the challenges of shipping samples to laboratories and shortens the time until results are available. A selective approach is defined as the use of antimicrobials only for cases that may benefit from them [2]. Selective treatment of clinical mastitis is preserved for mild (abnormal milk) and moderate cases accompanied with local signs in the udder [2]. Thirty percent or greater of clinical mastitis cases are culture-negative when sampled, for which the use of antimicrobials can be difficult to justify [3,4,5]. In addition, antimicrobial treatment of clinical mastitis cases caused by *E. coli* did not improve clinical outcomes [6]. However, extended treatment with intramammary antimicrobials was shown to be advantageous for clinical mastitis cases caused by environmental *Streptococci* [7,8] and *Staphylococcus aureus* [7,9]. Implementation of a selective treatment approach can reduce the use of antimicrobials on a given farm (i.e., no treatment for cows with no-growth or Gram-negative bacteria) and might have the potential to improve bacteriological cure (i.e., extended treatment for *Streptococcus uberis* and *Staphylococcus aureus*). Therefore, diagnostic accuracy is crucial for on-farm culture systems to be efficacious compared with a blanket treatment approach. A study comparing four commercial on-farm culture methods concluded that one test kit (Accumast^®^) was superior [10].

Therefore, the objectives of this study were (1) to determine if a treatment protocol for clinical mastitis (CM) based on on-farm culture results using Accumast^®^ (SELECT) led to similar clinical outcomes compared to blanket treatment (BT) of all cows with CM immediately, and (2) if such a protocol helped to reduce antimicrobial use.

## 2. Results

### 2.1. Descriptive Data

During the study period a total of 520 cases of CM were observed. Cows with severe mastitis (*n* = 44) and those with culture results considered contaminated (*n* = 8; in the SELECT group) were excluded. A sample was considered contaminated when the number of colony morphologies was greater than three, unless an obvious pathogen (clear predominance of one type of colony morphology) was noticeable. The remaining 468 cases were used for final analyses (BT = 236; SELECT = 232).

Parity distribution was 11.5% (55/468), 28.2% (132/468), and 60.0% (281/468) for first, second, and third lactation and greater. The overall mean and median days in milk (DIM) at enrolment were 134 d (±89.2) and 113 d (interquartile range 66–187 d), respectively. Fifty-four percent of cows (252/473) were experiencing their first mastitis event of the current lactation. Distribution of these descriptors by treatment group can be seen in Table 1.

In the SELECT group, there were 16.8% (39/232) cases with no growth, 13.4% (31/232) with a Gram-negative culture result, 40.0% (93/232) with *Streptococcus uberis*, and 29.7% (69/232) with a Gram-positive culture result other than *Streptococcus uberis*. Therefore, 30.1% (70/232) of CM cases were not treated with antimicrobials after enrolment.

### 2.2. Effect of Treatment Protocol

#### 2.2.1. Effect on Test-Day Milk Yield

The average test-day milk yield post-CM for all cows was 35.7 kg/d (*n* = 430). Full data existed for 187 BT cows and 202 SELECT cows. Milk yield was affected by parity (*p* = 0.013), milk yield before CM (*p* = 0.001), and days in milk (*p* = 0.001). Treatment had no effect on milk yield (*p* = 0.535; BT = 34.4 ± 0.78 kg/d; SELECT = 35.0 ± 0.77 kg/d). Cows in parity 2 (*p* = 0.03; 36.7 ± 0.88 kg/d) and 3 or greater (*p* = 0.11; 35.8 ± 0.64 kg/d) produced more milk compared with cows in parity 1 (31.6 ± 1.5 kg/d). There was no difference between parities 2 and 3 or greater.

#### 2.2.2. Effect on Linear Score

The average linear score (LS) post-CM for all cows was 4.6 (*n* = 430). Full data existed for 187 BT cows and 202 SELECT cows. Linear score was affected by LS before CM (*p* = 0.001). Parity tended to affect LS (*p* = 0.100). Treatment had no effect on LS (*p* = 0.284; BT = 4.4 ± 0.17; SELECT = 4.6 ± 0.17). Cows in parity 2 (*p* = 0.83; 4.7 ± 0.20) and 3 or greater (*p* = 0.36; 4.8 ± 0.14) had an increased LS compared with cows in parity 1 (4.1 ± 0.32). There was no difference between parity 2 and 3 or greater.

#### 2.2.3. Effect on Probability of Survival at 30 d

Of cows with full data, 7.7% (*n* = 36; BT = 21; SELECT = 15) did not remain in the herd at this time point. No significant difference was detected in odds of survival 30 d post-CM between the treatment groups (odds ratio (OR) = 0.74; 95% CI 0.37–1.47; *p* = 0.198). No other effects were significant in the model.

#### 2.2.4. Effect on Probability of Survival at 60 d

Of cows with full data, 15.6% (*n* = 73; BT = 45; SELECT = 28) did not remain in the herd at this time point. Cows in the SELECT group had reduced odds of being culled within 60 d post-CM (*OR* = 0.54; 95% CI = 0.31–0.93; *p* = 0.027). Days in milk at mastitis diagnosis (*p* = 0.010) also had a significant effect on culling risk within 60 d post-CM. Parity (*p* = 0.082) and number of mastitis events (*p* = 0.100) tended to affect culling risk within 60 d post-CM. Results are summarized in Table 2.

#### 2.2.5. Effect on Hospital Days

Hospital Days data were available for 463 CM cases (BT = 233; SELECT = 230). Treatment had no effect on hospital days (*p* = 0.786; BT = 6.6 ± 0.33 d; SELECT = 6.7 ± 0.33 d). Parity had an effect on hospital days. Cows in parity 3 or greater spent more time in the hospital pen (7.4 ± 0.27 d) compared with cows in parities 1 (*p* = 0.085; 6.6 ± 0.61 d) and 2 (*p* = 0.014; 6.2 ± 0.39 d). There was no difference between cows in parities 1 and 2 (*p* = 0.980).

## 3. Discussion

Implementation of a pathogen-based treatment protocol for mild and moderate CM cases resulted in no significant difference in post-event milk production, LS, survival to 30 d, and days spent in the hospital compared with a blanket therapy protocol. Surprisingly, there was an advantage regarding survival to 60 d for the selective treatment protocol. No-growth and Gram-negative cases determined by the Accumast^®^ system in the SELECT group were only treated with a non-steroidal anti-inflammatory drug (NSAID). These cases accounted for 30.1% of the CM cases.

The number of cases treated with antimicrobials in our study (69.9%) seems high compared with other clinical trials evaluating the effectiveness of selective treatment of CM relative to blanket treatment (Lago et al., 2011 [4]: 44%; MacDonald et al., 2011 [11]: 60%; Lago et al., 2016 [12]: 46%; Lago et al., 2016 [13]: 28%; Vasquez et al., 2017 [14]: 32%). The reason for this remains speculative. It might be affected by the overall prevalence of “no-growth” and Gram-negative pathogens in a herd. The predominant pathogen in our study being *Streptococcus ueris* (39.6%) resulted in a greater percentage of cows with extended antimicrobial therapy (5d vs. 3d). The prevalence of *Streptococcus uberis* in our study seems rather high compared with other recent studies (Lago et al., 2011 [4]: 14%; MacDonald et al., 2011 [11]: 21%; Lago et al., 2016 [12]: Vasquez et al., 2017 [14]: 32%). In other studies, coliform bacteria were the predominant pathogens (Lago et al., 2011 [4]: 24%; Vasquez et al., 2017 [14]: 34%), which were not treated with antimicrobials. Cultures with “no growth” accounted for 16.9% in our study. This seems rather small in comparison with other studies using on-farm cultures (Lago et al., 2011 [4]: 34%) or laboratory cultures (Vasquez et al., 2017 [14]: 33%). Another reason might be the diagnostic accuracy of an on-farm test to identify different pathogens. The accuracy of the Accumast^®^ system in identifying *E. coli* and no growth was 96.8% and 85.2%, respectively [10]. These results might differ when an on-farm culture system is used by farm personnel rather than trained laboratory technicians, as shown recently [15]. In that study, experience in milk microbiology substantially improved interpretation of on-farm culture results, indicating that the observers’ experience is crucial to facilitate appropriate management decisions when adopting a selective treatment protocol. We did not evaluate diagnostic accuracy in our study, but before the start of the study we trained farm personnel extensively in the use and interpretation of the Accumast^®^ system.

The current study did not find any differences in milk production and LS post-CM. These results are in agreement with previous studies comparing blanket treatment versus a selective treatment approach (no-growth and coliform-positive cows did not receive antimicrobials) using either an on-farm culture [4] or a laboratory culture system [14].

We observed no difference in days spent in the hospital pen (BT 6.6 d vs. SELECT 6.7 d). This is also in agreement with two recent trials (Lago et al., 2011 [4] BT 5.9 d vs. SELECT 5.2 d; Lago et al., 2016 [12] 6.7 d vs. 7.1 d). Two other previous studies observed a reduction in hospital days for a selective treatment protocol (Lago et al., 2016 [13] BT 6.7 d vs. SELECT 5.7 d; Vasquez et al., 2017 [14] BT 8.8 d vs. SELECT 5.8 d). Results are difficult to compare based on different treatment protocols (i.e., antimicrobial drugs with or without NSAID) and their resulting milk withholding time. In our study, all cows received an NSAID with a 5 d milk withholding time at enrolment, irrespective of the treatment group. In the two studies describing a beneficial effect on hospital days, no NSAID was administered at enrolment. It has been shown, however, that NSAID treatment in cows with CM positively affects clinical cure rate, milk production, somatic cell count, and culling risk [8,16,17,18].

Based on laboratory culture results in this particular herd, obtained before the start of the trial, a large proportion of cows with CM cases were positive with *Streptococcus uberis*. Therefore, we decided to implement an extended intramammary treatment protocol (5 vs. 3 d) for cows with *Streptococcus uberis* in the SELECT group and for cows with a first CM case in the BT group. An extended treatment with intramammary antimicrobials was shown to be advantageous regarding bacteriological cure for clinical mastitis cases caused by environmental streptococci (Oliver et al., 2004 [7]: Ceftiofur; Gillepsie et al., 2002 [8]: Pirlimycin). We decided to use a combination of Cefalexin and Kanamycin as a first choice. The extended treatment regime obviously affects days spent in the hospital pen, due to an extended milk withholding time.

Survival in the herd to 30 d post CM was not affected by treatment. This is in agreement with Lago et al. [4] and Vasquez et al. [14]. Survival in the herd to 60 d post CM, however, was improved in the SELECT group (OR = 0.54 for culling within 60 d). While the reason for this observation cannot be elucidated with our study design, it is, however, in agreement with a previous study, where removal from the herd within 21 d was reduced in the SELECT group compared with the BT group (OR = 0.48; [13]). In that particular study, only environmental streptococci were treated with antimicrobials in the SELECT group. We speculate that an immediate antibiotic treatment of all sick animals, as implemented in the BT group, might affect management decisions for herd removal later on. This should be investigated in the future. In our study, parity and number of CM events affected survival in the herd to 60 d, which is also in agreement with previous studies [14,19].

One drawback of this study was that we had no information on days to clinical cure and laboratory culture results from CM cases for both treatment protocols. Serial cultures after CM to determine bacteriological cure can be considered the gold standard in research trials to assess treatment efficacy. However, initial cultures can return negative results, limiting the ability to determine cure. Follow-up samples may also result in a different pathogen or contamination. Indirect assessment of treatment efficacy using milk yield, LS post CM and survival in the herd has been utilized as a reliable alternative [20].

Appearance of normal milk may not constitute elimination of the infection, but most producers rely on this observation for the decision-making process. Instead, we used days spent in the hospital pen as a proxy for cure. Days out of the tank is an important attribute, as it has been linked to the economics of selective treatment approaches for mild and moderate CM cases [21].

The authors recognize that trials using multiple herds may provide additional data that accounts for variation among herds. This is clearly a limitation of the current study, as this study population is not representative of an external population. Certainly, one should not generalize the results of this study to dairy farms that do not have *Streptococcus uberis* as the dominating mastitis causing pathogen. Nevertheless, *Streptococcus uberis* is considered a major pathogen on modern dairy farms [22].

Cows with severe cases of CM were excluded. Research has shown that the use of systemic antibiotics is beneficial for the treatment of septicemia that occurs in many cows affected with severe mastitis [22]. Therefore, these cows were not assigned to the selective treatment protocol.

Cows with repeated cases of CM were assigned to treatment with Cefoperazon in both BT and SELECT. We tested common mastitis pathogens on the farm for antimicrobial resistance before the start of the trial. Most of the Gram-positive pathogens were sensitive to Cefoperazon. There was no difference in the percentage of repeated cases between BT and SELECT. Therefore, we do not expect that the results of this trial are biased by this treatment.

## 4. Materials and Methods

### 4.1. Study Animals

Clinical mastitis cases were assessed for inclusion at a 2600 Holstein cow commercial dairy in Mecklenburg-Vorpommern, Germany, between September 2017 and December 2017, under Institutional Animal Care and Use Committee approval. This farm was chosen due to its large herd size, a monthly incidence of 5 to 6% CM, the availability of reliable health records and its willingness to participate. This farm used Dairy Herd Improvement Association services, which included monthly somatic cell count and milk weights. Health records included treatment, treatment pen moves, and culling data.

### 4.2. Case Definition

Each CM case was detected by trained on-farm employees by observing abnormalities in milk, such as changes in consistency and color, or udder signs, including hard, swollen, or red quarters. Farm personnel were trained using standard operating procedures provided by the Clinic of Animal Reproduction. Cows exhibiting severe symptoms such as depression, anorexia, dehydration, or fever received systemic antimicrobials and anti-inflammatories and were excluded from the study. Other exclusion criteria included treatment with antimicrobials or anti-inflammatories in the previous 15 d and impending sale of the animal. Subsequent cases from an individual cow were included. A cow was not excluded if it had had one or more cases of mastitis before enrollment.

### 4.3. Sample Collection and Treatment Assignment

Using a sterile technique, a milk sample was collected from each affected quarter into a milk culture tube. The tube was labeled and promptly placed in a 5 °C refrigerator. Quarter and date entering hospital pen were recorded in Dairy Comp 305 (DC305; Valley Agricultural Software, Tulare, CA, USA). Milk sampling, inoculation of plates, reading out and treatment of cows were conducted by farm personnel based on standard operating procedures provided by the Clinic of Animal Reproduction. Milk samples were cultured using Accumast^®^ (FERA Animal Health LCC, Ithaca, NY, USA). The diagnosis of mastitis-related pathogens or groups of bacteria was carried out according to the manufacturers’ recommendations. Briefly, milk samples were plated onto Accumast^®^ using a sterile cotton swab. Before application into each of the three sections of Accumast^®^ the swab was immersed in the milk sample. Plates were incubated at 37 °C for 24 h and read on-farm by farm personnel according to the flowchart provided for on-farm diagnosis of mastitis pathogens identifiable by Accumast^®^. The Accumast^®^ system uses three selective chromogenic media to identify specific bacteria or group of bacteria. Accumast^®^ identifies *Staphylococcus aureus*, *Staphylococcus* spp., *Streptococcus* spp., *Enterococcus* spp. or *Lactococcus* spp. (EL group), *Klebsiella* spp., *Enterobacter* spp. or *Serratia* spp. (KES group), *E. coli*, Gram-negative bacteria other than *E. coli* or KES, and milk samples with no bacterial growth.

Cows in the herd were randomly assigned by DC305 to either the blanket therapy group or the pathogen-based treatment group. If a cow was enrolled a successive time, the same treatment group was assigned.

### 4.4. Treatment Groups

**Figure 1:**
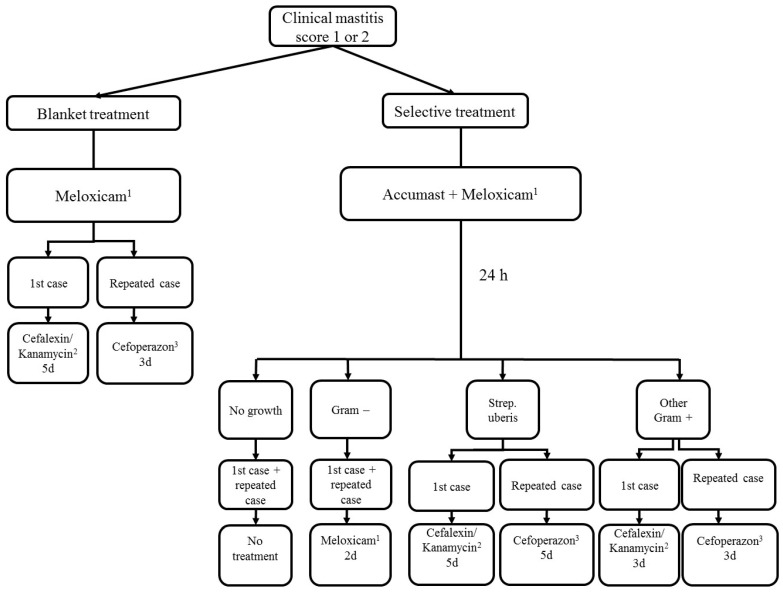
Schematic presentation of the treatment protocols assigned to blanket treatment and selective treatment. ^1^ Metacam, Boehringer-Ingelheim, Ingelheim, Germany. ^2^ Ubrolexin, Boehringer-Ingelheim, Ingelheim, Germany. ^3^ Peracef, Zoetis, Berlin, Germany.

Irrespective of severity score or treatment group, all cows received 0.5 mg/kg meloxicam (Metacam, Boehringer-Ingelheim, Ingelheim, Germany) on the initial day of CM diagnosis. Treatment of mild or moderate cases of CM is summarized in Figure 1 for cows in the SELECT and BT group.

#### 4.4.1. Blanket Therapy Group

Cows with the first case of CM received 200 mg Cefalexin and 100,000 I.U. Kanamycin (Ubrolexin, Boehringer-Ingelheim, Ingelheim, Germany) in the blanket therapy (BT) group, immediately after enrollment. The treatment was repeated once every 24 h for 5 d according to label directions. Cows with a subsequent case of CM received 100 mg Cefoperazon (Peracef, Zoetis, Berlin, Germany). The treatment was repeated once every 24 h for 3 d according to label directions.

#### 4.4.2. Pathogen-Based Treatment Group

In the pathogen-based (SELECT) treatment group, results from the on-farm culture system were transferred to DC305. Cows were automatically assigned to one of the following treatment options based on the culture results: (1) cows with no growth received no further treatment, (2) cows positive with Gram-negative bacteria received two additional treatments with 0.5 mg/kg meloxicam 24 h apart, (3) cows with a first case of *Streptococcus Uberis* or environmental *Streptococci* received one 200 mg Cefalexin and 100,000 I.U. Kanamycin (Ubrolexin, Boehringer-Ingelheim, Ingelheim, Germany) every 24 h for 5 d. Cows with a subsequent case of *Streptococcus uberis* or environmental *Streptococci* received 100 mg Cefoperazon (Peracef, Zoetis, Berlin, Germany) every 24 h for 5 d, (4) cows with a first case of other Gram-positive bacteria received 200 mg Cefalexin and 100,000 I.U. Kanamycin (Ubrolexin, Boehringer-Ingelheim, Ingelheim, Germany) every 24 h for 3 d. Cows with a subsequent case of *Streptococcus uberis* or environmental *Streptococci* received 100 mg Cefoperazon (Peracef, Zoetis, Berlin, Germany) every 24 h for 3 d.

All CM cows remained in the hospital pen until milk withdrawal times were met and milk returned to normal visual appearance. Entrance and exit dates were recorded by on-farm personnel. Any cow that graduated to severe clinical signs exited the trial and was treated systemically according to veterinary recommendations.

### 4.5. Treatment Outcomes

Cows in the study were followed up for post-treatment milk production, post-treatment linear score, survival in the herd, and days in hospital pen (number of days between entrance and exit into and from this pen). Linear score is a transformation of somatic cell count (SCC), calculated as [ln(SCC/100)/ln(2)] + 3 [23]. Post-treatment LS and milk production were obtained from test day data between 8 and 43 d post-CM event. All values and dates were retrieved from the on-farm management software.

### 4.6. Data Collection and Statistical Analyses

All statistical analyses were performed using SPSS for Windows (version 22.0, SPSS Inc., IBM, Ehningen, Germany). Cow was the experimental unit. The effects of selected explanatory variables on post-treatment outcomes were analyzed using the GENLINMIXED procedure in SPSS for continuous variables (i.e., LS, milk yield, hospital days) and dichotomous outcomes (i.e., survival). Continuous explanatory variables included days in milk at CM event, milk production and LS before treatment (8 to 43 d before the event). Explanatory categorical variables tested were mastitis event (first, second, or third or greater) and parity (first, second, or third or greater). Each parameter considered for the mixed model was separately analyzed in a univariable model. Only parameters resulting in univariable models with *p* ≤ 0.10 were included in the final mixed model. Selection of the model that best fit the data was performed using a backward stepwise elimination procedure that removed all variables with *p* > 0.10 from the model. Regardless of significance level, treatment (BT vs. SELECT) was forced to remain in the model. Five models were used, describing the treatment effect on (1) milk yield, (2) LS, (3) the odds of survival at 30 d, (4) the odds of survival at 60 d, and (5) the average number of hospital days. Not all included cows had complete LS or milk yield data, as some cows experienced their CM event in early lactation with no prior test day or experienced the CM event late in lactation with no post-CM test day. Cows were excluded from hospital days analysis if pen moves were incomplete. An animal was not included in a model if it was missing a data point for a parameter offered to the model. Therefore, the number of animals was indicated for each analysis. Variables were declared to be significant when *p* ≤ 0.05. A statistical tendency was declared when *p* was between 0.05 and *p* ≤ 0.10.

## 5. Conclusions

Results from the present study suggest that a delayed treatment of mild and moderate CM cases using a selective treatment approach with on-farm culture resulted in no negative effects on milk yield, LS, days spent in the hospital, and culling risk two months following the CM event. However, one-third of the CM cases was not treated with intramammary antimicrobials. Therefore, a selective treatment approach provides an opportunity to reduce antimicrobial use on dairy farms without affecting health, performance, and welfare of animals.

## Figures and Tables

**Table 1:** Cow level descriptors for each treatment group.

Item	Treatment Group		*p*-Value
	Blanket Therapy (*n* = 236)	Selective Treatment (*n* = 232)	
Parity, no.			0.368
1st	28 (11.8%)	27 (11.6%)	
2nd	73 (30.9%)	59 (25.4%)	
≥3rd	135 (57.3%)	146 (63.0%)	
DIM	128.5	139.8	0.164
Mastitis event, no. ^1^			0.172
1st	117 (49.6%)	135 (58.1%)	
2nd	70 (29.7%)	56 (24.3%)	
≥3rd	49 (20.7%)	41 (17.6%)	
Milk yield, kg	36.9	37.8	0.495
Linear score	3.6	3.4	0.408

^1^ Cow-level mastitis event at time of enrolment for the current lactation.

**Table 2:** Logistic regression for survival to 60 d after clinical mastitis event ^1^.

Parameter	Description	Estimate	SE	*p*-Value	Odds Ratio	95% CI
Intercept		−2.32	0.49	0.001		
Treatment	Blanket treatment	Referent				
	Selective treatment	−0.61	0.27	0.027	0.54	0.31–0.93
Parity	1	Referent				
	2	−0.46	0.49	0.353	0.63	0.24–1.67
	≥3	0.30	0.43	0.479	1.35	0.58–3.15
Mastitis event	1	Referent				
	2	0.24	0.33	0.468	1.27	0.66–2.43
	≥3	0.72	0.34	0.035	2.06	1.05–4.04
Days in milk		0.01	0.01	0.010	1.01	1.01–1.02

**^1^** Probability modeled was culled at 60 d after a clinical mastitis event.

## Data Availability

The data presented in this study are available on request from the corresponding author. The data are not publicly available due to ownership of the farm.
